# Qualitative Behavioural Assessment of emotionality in pigs

**DOI:** 10.1016/j.applanim.2012.04.004

**Published:** 2012-07

**Authors:** Kenneth M.D. Rutherford, Ramona D. Donald, Alistair B. Lawrence, Françoise Wemelsfelder

**Affiliations:** Animal Behaviour and Welfare, Animal and Veterinary Sciences Research Group, SAC, West Mains Rd., Edinburgh, EH9 3JG, Scotland, United Kingdom

**Keywords:** Qualitative Behavioural Assessment, Free Choice Profiling, Welfare, Pigs, Open field, Elevated plus maze

## Abstract

Scientific assessment of affective states in animals is challenging but vital for animal welfare studies. One possible approach is Qualitative Behavioural Assessment (QBA), a ‘whole animal’ methodology which integrates information from multiple behavioural signals and styles of behavioural expression (body language) directly in terms of an animal's emotional expression. If QBA provides a valid measure of animals’ emotional state it should distinguish between groups where emotional states have been manipulated. To test this hypothesis, QBA was applied to video-recordings of pigs, following treatment with either saline or the neuroleptic drug Azaperone, in either an open field or elevated plus-maze test. QBA analysis of these recordings was provided by 12 observers, blind to treatment, using a Free Choice Profiling (FCP) methodology. Generalised Procrustes Analysis was used to calculate a consensus profile, consisting of the main dimensions of expression. Dimension one was positively associated with terms such as ‘Confident’ and ‘Curious’ and negatively with ‘Unsure’ and ‘Nervous’. Dimension two ranged from ‘Agitated’/‘Angry’ to ‘Calm’/‘Relaxed’. In both tests, Azaperone pre-treatment was associated with a more positive emotionality (higher scores on dimension one reflecting a more confident/curious behavioural demeanour) than control pigs. No effect of drug treatment on dimension two was found. Relationships between qualitative descriptions of behaviour and quantitative behavioural measures, taken from the same recordings, were found. Overall, this work supports the use of QBA for the assessment of emotionality in animals.

## 1 Introduction

The assessment of affective states in animals is a critical component of animal welfare research. In recent years a variety of approaches have been applied to address this (e.g. appraisal theory: Boissy et al., 2007a; cognitive bias: Mendl et al., 2009). Qualitative Behavioural Assessment (QBA) is one such method. QBA is a whole-animal approach, and the underlying premise is that human observers can integrate perceived behavioural details and signals to judge an animal's behavioural expression, using qualitative descriptors (e.g. relaxed, anxious) that reflect the animals’ affective (emotional) state (Wemelsfelder, 1997, 2007). QBA allows for a scientific basis to be applied to the characterisation of behavioural expressions of animals in terms of their affective experience. A number of studies in pigs (Wemelsfelder et al., 2001, 2001, 2009) and other species (Rousing and Wemelsfelder, 2006; Napolitano et al., 2008; Minero et al., 2009; Walker et al., 2010) have shown that data generated from such observations are reliable and repeatable, and correlate to assessments of the animal's physical behaviour. As such, there is increasing indication that QBA can be a valuable methodology for assessing behavioural expression in farm animals under field conditions (Brscic et al., 2009; Wemelsfelder and Millard, 2009), and more broadly, that qualitative rating scales can have useful practical applications in assessing animal behaviour (Meagher, 2009). A recent review of methodologies that might be used to assess positive welfare states in cattle concluded that QBA was ‘the most promising’ assessment methodology (Napolitano et al., 2009). Boissy et al., 2007b also noted that QBA represented one of the most immediately applicable methodologies for assessing positive emotions in animals. A report from the UK Farm Animal Welfare Council (FAWC, 2009) on the future of animal welfare research emphasised the importance of including consideration of positive welfare states and the role that QBA could play in assessing these.

For QBA, like any new measurement tool, the on-going process of validation is critical. Validation is a process of iterative hypothesis testing; as more is learnt about the construct putatively underlying the measurement scheme, new predictions can be generated and tested against further observation (Streiner and Norman, 2008). In this broad view, the validity of a measurement tool is never completely proven; successive new data influence the degree of confidence that can be placed on inferences about individuals based on their scale scores. Consequently, no one experiment can be carried out which ultimately proves the theory underlying the relationship between the tool and the construct it is thought to measure (Streiner and Norman, 2008). To date QBA has stood up well to the process of validation testing from the perspective of its reliability and relationship to quantitative measures of behaviour. An important ongoing question is whether and how QBA outcomes relate to physiological and neurobiological parameters, an issue considered crucial by many scientists in demonstrating the biological validity of QBA. A promising start in addressing this question was made by Stockman et al. (2011), who found QBA outcomes to correlate well to a number of physiological stress indicators in cattle during transport.

In this study, QBA was applied to video recordings taken from young pigs exposed to either an open field (OF) or Elevated-Plus-Maze (EPM) test with or without pre-treatment with Azaperone. Azaperone is a butyrophenone neuroleptic drug currently licensed for pigs (to prevent aggression and stress, e.g. Tan and Shackleton, 1990). Although primarily used as a sedative, at low doses Azaperone has been found to reduce emotionality in sheep tested in an open field test (Hughes et al., 1977) and to increase inter-individual distance and lower shade preference when given to sheep before testing in a novel environment (Madsen et al., 1980). More broadly Azaperone is thought to act on the brain to make animals indifferent to their surrounding environment (Dantzer, 1977; Pascoe, 1986). Studies have shown that Azaperone causes quantitative changes in pig behaviour that could be interpreted as indicating an anxiolytic effect (Donald et al., 2010, 2011). Behavioural tests such as the EPM and OF are commonly used to examine states of anxiety and fear in many species, including pigs, yet their validity is often only poorly established in farm animals (Forkman et al., 2007). The work presented here was part of a series of experiments which aimed to examine the validity of using OF and EPM behavioural measures to assess emotionality in pigs. The specific aim of the current study was to test how QBA judgements of behavioural expression differed when observers viewed footage of pigs whose emotional state had been putatively altered through prior treatment with Azaperone compared to control pigs treated with saline.

## 2 Methods

### 2.1 Animals

This study was conducted following ethical approval by the Animal Experiments Committee at SAC, and under UK Home office licence. Two separate experiments were carried out examining the effects of the drug Azaperone on pig behaviour in either an open field (OF) or elevated plus-maze (EPM) test. Quantitative behavioural measures from the OF observations assessed here have been previously published (Donald et al., 2011). In both experiments, piglets were born in standard farrowing crates and weaned into pre-allocated smaller groups of 4–6 (balanced as far as possible for sex and weight) at around 4 weeks of age. They were then moved to pens (2.85 m × 1.85 m) with concrete floors and deep straw bedding. All animals had *ad libitum* access to feed and water and pens were cleaned daily and replenished with fresh straw.

### 2.2 Experiment 1: open field

In Experiment 1, the subjects were 12 (7 males, 5 females), 38.0d (SD = 1.0 d) old Landrace × Large White pigs taken from 3 litters. Each pig was tested in the OF twice (exposure one and exposure two) for 10 min in a cross-over design, once with a (1 mg/kg) pre-exposure intra-muscular injection of Azaperone (Stresnil: Janssen Animal Health (Elanco), Brussels, Belgium) and once with a pre-exposure intra-muscular injection of an equivalent volume of saline. The first and second exposures were 3 d apart and the order in which pigs were tested was maintained on both occasions. Following injection in the home pen, pigs were left undisturbed with littermates for 20 min before being observed in the test apparatus in an adjacent room. To start the test, each pig was picked up and carried to an adjacent room where it was placed in the open field. The open field arena (1.84 m × 1.89 m) had 0.90 m high solid walls, a concrete floor, and was provisioned with two unfamiliar objects, an orange ball (65 cm circumference) and a feeder (21.5 cm  × 9.5 cm × 9.0 cm). The arena was washed down with water between pigs to reduce odour from the preceding pig. During the test, pig behaviour was recorded onto a digital video camera for subsequent analysis. Two 1 min periods during the test (min 1 and 8) were selected from each recording for subsequent qualitative analysis. Min 1 was chosen to show the initial reaction to the test and min 8 was chosen as an arbitrary point towards the end of the test.

### 2.3 Experiment 2: elevated plus-maze

Subjects were 28 (16 males, 12 females), 57.5 d (SD = 0.5 d) old Landrace × Large White pigs taken from 3 litters. Each pig was tested once in the EPM for a period of 5 min. Half the pigs (*n* = 14) received a pre-exposure intra-muscular injection of Azaperone (1 mg/kg) and half (*n* = 14) were given an equivalent volume of saline. The EPM was a version of the one described by Andersen and colleagues (Andersen et al., 2000a, 2000b). The plus-shaped apparatus was elevated (65 cm) from the ground, and consisted of 4 arms (192 cm × 80 cm), joined by a central octagonal-shaped platform. Two arms had transparent acrylic glass sides (closed arms) with no roof, and two arms had open sides. The edges of the two open arms had a barrier 9 cm along the edge and the ends of the arms were blocked off to prevent pigs falling off the apparatus. Padded mats were placed on the ground around the open arms in case pigs did fall or jump from the EPM. The sides of the closed arms were 60 cm high. Black rubber covered the floor of the central platform and four arms. A thick covering of wood-shavings covered the rubber floor and excreta were removed and wood-shavings were replaced between successive pigs.

Twenty minutes after injection with saline or Azaperone in the home pen, the pigs were picked up and carried to an adjacent room where they were placed through a slide door that led directly to the central platform of the EPM. The door was slid shut and the experimenters immediately left the room. Pig behaviour was again recorded onto a digital video camera for subsequent analysis. Two 1 min periods (min 1 and 5) were selected from each recording for subsequent qualitative analysis. As with the OF min 1 was chosen to show the initial reaction to the test and min 5 was chosen as the last minute of the shorter EPM test.

### 2.4 Qualitative Behavioural Assessment

Participating observers were 12 MSc students in Applied Animal Behaviour and Animal Welfare, who all had a general understanding of animal behaviour, but no specific expertise in pig behaviour. These observers scored pig behavioural expression in the OF and EPM following protocols as developed for Free Choice Profiling (FCP) methodology (Wemelsfelder et al., 2000, 2001). The first stage of the FCP method requires that observers generate their own descriptor lists for use in subsequent assessments. For this 16 demonstration clips (1 min long), deemed to be representative of the range of different behavioural styles and demeanours observed in the tests, were selected (eight for each apparatus) and shown to the observers. All demonstration clips and the subsequent study clips included sound. The 16 clips were shown to observers in two batches with a 10 min break between each batch. Each batch consisted of both OF and EPM footage but no more than two clips of the same apparatus were played in succession. The reason for showing recordings of both types of test in a single term generation session was that a single vocabulary would be produced that was applicable across both tests. After each individual clip, observers were given 2 min to write down as many descriptive terms for the observed pig as they thought were needed to adequately characterise that pig's behavioural expressions. All the terms generated in this way were then collated for each observer and used to create each observer's individual list of terms. When individual observers used both positive and negative antonyms (e.g. “confident” and “unconfident”, “comfortable” and “uncomfortable”), only the positive term was kept for use in subsequent scoring. The number of terms generated by each observer ranged from 11 to 43. For each observer's score sheet, the terms were arranged so that successive terms had contrasting meaning with (as far as possible) similar terms being listed further apart.

In the second stage of the FCP process, the observers scored the full set of clips, each using their individual list of previously generated terms. This scoring took place over four sessions on different days. On each day, observers were shown a batch (11 or 12 clips) of OF clips and a separate batch (13 or 14 clips) of EPM clips, with a break between batches. The order of OF and EPM batches was rotated each day. Clips were allocated to a session so that sessions were as far as possible balanced for whether pigs were drug-treated or not, by litter group, and recording time within the observation. The open field clips were also balanced for order of testing since the pigs were tested in the apparatus twice. Following viewing of each individual clip, observers were asked to quantify, for each term, the degree of expression shown by the pig, by marking a vertical line on a 125 mm visual analogue scale, ranging from minimum to maximum possible expression. Observers were unaware of any prior drug or other treatment applied to the pigs. They were instructed that the purpose of the experiment was to investigate and compare behavioural expression in the 2 different types of test.

Prior quantitative assessment of pig behaviour in these tests (Donald et al., 2010, 2011) identified three main factors that change with Azaperone treatment: activity, vocalisations and exploration. To provide a comparison with the qualitative measures of behaviour, these same quantitative measures of behaviour (see Table 1 for a summary Ethogram) were recorded (using event logging software: Observer 5.0, Noldus Information Technology) from the same 1 min long clips used for QBA.

### 2.5 Data analysis

Data (distance from zero along the visual analogue scale in millimetres) for each observer's scoring of every one of their individual terms for each clip were analysed using Generalised Procrustes Analysis (GPA), as previously described in detail (Wemelsfelder et al., 2000, 2001, 2009). Briefly, GPA is a multivariate technique that identifies underlying patterns in data that do not consist of common fixed variables. The statistical process whereby this best-fit pattern, termed the consensus profile, is identified takes place independently of the meaning of individual terms used by observers. The Procrustes statistic is calculated quantifying the percentage of variation between observers (in their assessment of individual pigs) that is explained by the consensus. The statistical performance of the consensus profile above chance is calculated by comparing (using a one-sample *t*-test) the Procrustes statistic to the mean of a simulated distribution of 100 Procrustes statistics generated through 100 iterations of the analysis where the data is randomised in a different permutation each time. Significance values in that test of *P* < 0.001 or better can be taken as evidence that the consensus profile was not a methodological artefact and does represent a common pattern identified by observers. The Procrustes statistic can also be used to assess the degree of agreement between individual observers and the overall consensus profile. To do this, Procrustes statistics are calculated for all possible pairs of observers and Principal Coordinate Analysis is used to place all observers on a two-dimensional plot (known as the observer plot), along with a 95% confidence region defining the ‘normal population’ of observers. Principal Component Analysis is then used to reduce the many dimensions within the consensus profile to a smaller number of dimensions, which explain the majority of variation between observed animals. To allow for semantic interpretation of these main dimensions, the score for individual observer terms can be correlated with the overall dimension score (i.e. the more highly correlated an individual term is with a dimension, the more weight it has as a descriptor – positive or negative – for that dimension). This process is entirely post hoc to the computation of the consensus profile but allows identification of the individual terms that best describe the anchor points at each end of the main dimensions for purposes of interpretation.

Raw data from both OF and EPM tests were analysed together in a single merged GPA. Preliminary analysis found that the correlations between pigs in scores from separate or merged analyses were very high, indicating that the merged analysis did not alter the ranking of the pigs in the separate analyses. However, subsequent statistical analysis considered the two tests separately. The impact of prior drug treatment on QBA was examined using REML in Genstat (Genstat Release 10, VSN International Ltd., Hemel Hempstead, U.K.). For each apparatus, both recordings taken during a single test exposure were included in the analysis. For the EPM observations where Azaperone and saline treated pigs were tested as separate treatment groups, pig sex, drug treatment and clip time (first or second) were included as fixed effects and litter and pig were included as random terms in the model. For the open field observations where individual pigs were tested twice in a cross-over design, pig sex, test order, drug treatment and clip time were included as fixed effects, and litter and pig were included as random effects. For both tests, initial models included all possible interaction terms, but where these were found to be non-significant, they were removed from the model. Normality of the residuals was established by visual inspection of residual plots. All final quoted test statistics and significance values reflect the additional effect of that parameter on QBA dimensions after other effects had been statistically accounted for. The relationships between QBA scores and quantitative measures of behaviour from the same clips were explored by calculating Spearman rank correlations between quantitative and qualitative data.

## 3 Results

### 3.1 QBA dimensions

The consensus profile explained a significantly higher percentage of variation (Procrustes statistic: 49.73%) than the mean of 100 randomised analyses (mean ± SE Procrustes statistic: 25.27 ± 0.022; *t*_99_ = 109.87; *P* < 0.001), indicating that the consensus profile reflects an underlying attribute of the data not generated through chance. All but two observers, who were marginal outliers, fell within the 95% confidence region, reflecting a high level of inter-observer agreement in identifying underlying patterns within the data. Two main dimensions of expression were found that accounted for 45.7% and 23% of the variation, respectively. Assessment of the words positively or negatively associated with each dimension (Table 2) demonstrates the semantic coherence generated across observers (i.e. that the different individual observers use terms with similar meanings when viewing particular behavioural expressions). The terms most commonly associated with dimension one were confident and curious (positive) and unsure and nervous (negative) and those most commonly associated with dimension two were agitated, and angry (positive) and calm, and relaxed (negative). These terms were therefore used as labels to provide semantic understanding of the anchor points of each dimension.

### 3.2 Experiment one: open field

**Figure 1:**
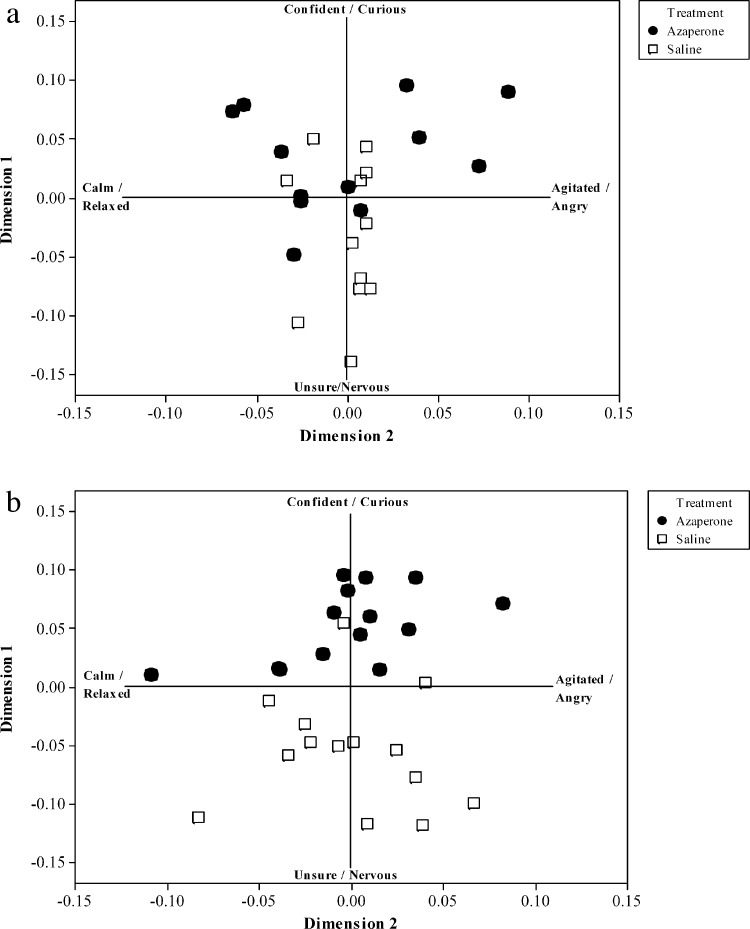
QBA analysis of pig behaviour in an open field test (a) or elevated plus-maze test (b) with or without prior treatment with Azaperone.

There was a highly significant effect of drug treatment on the pigs’ scores on dimension one (a continuum from nervous to confident) of the QBA consensus profile (*W* = 19.66, *P* < 0.001; Fig. 1a), with no effect on dimension two (a continuum from calm to agitated) (*W* = 0.00, *P* = 0.98). Treatment with Azaperone was associated with more positive behavioural expression (i.e. higher dimension one scores, reflecting a confident/curious behavioural demeanour). There was no impact on either dimension of pig weight (Dimension one: *W* = 3.33, *P* = 0.102; Dimension two: *W* = 0.04, *P* = 0.954) or sex (Dimension one: *W* = 0.06, *P* = 0.805; Dimension two: *W* = 1.45, *P* = 0.261), or the time point within the observation (Dimension one: *W* = 0.84, *P* = 0.366; Dimension two: *W* = 1.27, *P* = 0.268). There was a difference between exposure one and exposure two for dimension one (*W* = 10.99, *P* = 0.002); pigs were more confident/curious during the first exposure compared to the second. No such effect was seen on dimension two (*W* = 0.72, *P* = 0.402).

### 3.3 Experiment two: elevated plus-maze

There was a highly significant effect of drug treatment on the pigs’ scores on QBA dimension one but not dimension two (Dimension one: *W* = 34.98, *P* < 0.001; Dimension two: *W* = 0.16, *P* = 0.696; Fig. 1b). As with the OF test, pigs that received prior treatment with Azaperone were scored as being more confident/curious and therefore less nervous than those receiving a saline injection. Neither pig weight (Dimension one: *W* = 0.02, *P*= 0.88; Dimension two: *W*= 0.15, *P* = 0.703) or sex (Dimension one: *W* = 0.06, *P*= 0.816; Dimension two: *W*= 0.12, *P* = 0.734) impacted on scores on either dimension. Pigs were scored as being slightly more confident (Dimension one: *W* = 4.24, *P* = 0.05), but no more or less calm (Dimension two: *W* = 2.07, *P* = 0.63), at the end of the test than they were at the start.

### 3.4 Correlations between qualitative and quantitative assessments

The quantitative measures of activity (zones visited for EPM, squares crossed for OF) were both significantly positively correlated with pigs’ scores on dimensions one and two in both the OF (D1: *r* = 0.440, *P* = 0.002; D2: *r* = 0.359, *P* = 0.013) and EPM tests (D1: *r* = 0.562, *P* < 0.001; D2: *r* = 0.421, *P* = 0.002). The frequency of low grunts was positively correlated with scores on dimension two in both tests (OF: *r* = 0.435, *P* = 0.002, EPM: *r* = 0.449, *P* = 0.001). Grunting was also highly significantly negatively correlated with dimension one in the EPM test (*r* = −0.608, *P* < 0.001), but was not correlated with dimension one in the OF (*r* = 0.233, *P* = 0.115). For the EPM, there was no relationship between the time spent rooting in the substrate and either dimension (D1: *r* = 0.219, *P* = 0.115; D2: *r *= −0.08, *P* = 0.57). However, in the OF the time spent exploring the pen was highly positively correlated with dimension one (*r* = 0.487, *P* = 0.001), but not with dimension two (*r* = −0.208, *P* = 0.161).

## 4 Discussion

A Qualitative Behavioural Assessment (QBA) of grower pig behavioural expression in either an open field or elevated plus maze identified differences between pigs pre-treated either with the drug Azaperone or with saline. These data clearly show that QBA is sensitive to the putative experimental alteration of emotional state, achieved through this pharmacological manipulation. This is the first such demonstration and adds to the process of validation of QBA. The finding that observers, who were blind to the experimental treatment, were able to distinguish between pigs that had been given either saline or Azaperone, strongly supports the biological validity of QBA. The clear discrimination between drug-treated and control pigs is particularly striking since it was made on the basis of two short recordings of only 1-min duration, which were chosen strictly to adhere to particular time windows. In both the open field (OF) and elevated plus maze (EPM) test situations, Azaperone treated pigs were seen to be more confident and curious (and conversely less unsure and nervous) compared to saline treated pigs. No difference was seen on a second dimension which classified behavioural expression along a continuum from calm/relaxed to agitated/angry. This highlights the differences between the behavioural effects of low-dose Azaperone and those of more traditional anxiolytic drugs such as Diazepam (e.g. Andersen et al., 2000b; Donald et al., 2011). This may reflect the different pharmacological actions of Azaperone, which acts on the Dopamine system, and drugs such as Diazepam that act on the GABA-benzodiazepine-receptor complex. Azaperone appears to decrease fear/anxiety but replaces it with a more active behavioural phenotype than the calming effect associated with other anxiolytic drugs.

In the OF test experiment, pigs were used as their own controls in a cross-over design. QBA scores also suggested that pigs were more anxious during their second exposure to the OF test, a finding also reported for quantitative measures of behaviour when pigs were exposed to an OF test twice without any drug interventions (Donald et al., 2011). Although exposure to the open field is essentially harmless and animals will eventually habituate to repeat exposure to such short-term isolation, these data suggest that a second exposure may provoke an increased anxiety response. Behavioural responses in an open field test are the consequence of a complex mix of different motivations in the animal. Possibly, the apparent increased level of anxiety in the second exposure is a consequence of the changing balance of these different motivations. The decrease in exploratory behaviour in the second exposure (also seen by Donald et al., 2011), most likely due to the reduced novelty element, combined with the remaining negative components associated with isolation may produce a more anxious behavioural profile.

The use of specific drug manipulations has played an important part in the validation of quantitative measures of emotionality in tests such as the EPM or OF for rodents. The a posteriori assumption based on the prior use of QBA was that if it indeed provided a direct assessment of an animal's affective state then an alteration of affective state through a pharmacological manipulation should be reflected in altered QBA scores. The fact that this was clearly the case here supports the biological validity of QBA generally and more specifically as an outcome measure of emotionality in forced exposure tests such as the EPM and OF. However, as noted in the introduction, validation should be regarded as a process of iterative hypothesis testing rather than something with a fixed end point (Streiner and Norman, 2008). From this perspective, further exploration of the biological basis to QBA outcomes is warranted.

The outcome of this study does not necessarily imply that QBA is better at discriminating the effects of Azaperone than normal quantitative measures. However, the work presented here may aid the interpretation of quantitative behavioural measures of emotionality in the EPM and OF tests for pigs. The analysis of the relationships between qualitative and quantitative behavioural data further highlights the value of an integrative approach to interpreting behaviour (see also Rousing and Wemelsfelder, 2006; Walker et al., 2010). In both tests, total physical activity was positively correlated with scores on both dimensions one (nervous to confident) and two (calm to agitated). Since a high score on dimension one indicates a relatively positive state (confident/curious) and a high score on dimension two might be thought of as indicating a relatively negative state (agitated/angry) this shows how quantified physical activity may reflect both positive and negative aspects of emotional experience, or a combination of these aspects. High levels of physical activity could signify the confidence and curiosity of animals exploring an arena, the agitation of animals attempting to escape, or a combination of both such expressions. Thus measurements of physical activity per se appear to have limited value in informing assessments of pig emotionality in such tests. Alternatively, the frequency of low grunts was highly negatively correlated with dimension one in the EPM and positively correlated with dimension two (i.e. animals that grunted a lot were qualitatively scored as either unsure/nervous or agitated/angry) in both the EPM and OF tests. This indicates that grunting was more reliably associated with negative affective states in either test, reflecting the wider utility of measuring animal vocalisations in the assessment of affective states (Düpjan et al., 2008). Indeed, since observers had access to audio from the video recordings grunting may have been used as an indicator of emotionality by observers.

## 5 Conclusion

In conclusion, the demonstration here of the sensitivity of qualitative assessment to pharmacologically altered neurophysiological state in pigs supports the application of QBA within animal welfare assessments (where assessing negative and positive emotionality is of critical importance (Boissy et al., 2007b; FAWC, 2009)). This work supports the guiding hypothesis that QBA, rather than consisting of unfounded projections of human emotion, is empirically grounded in the observation of behavioural signs that, according to previously agreed criteria (i.e. administration of anxiolytic drugs), reflect an animal's emotional state. As such, QBA has substantial potential to aid animal welfare assessment, in combination with other approaches, whether as part of experimental studies or on-farm welfare assessment.

## Figures and Tables

**Table 1:** Ethogram of measures used for quantitative assessment of behaviour in the 1-min observations used for QBA.

	Open field	Elevated plus maze
Activity	Squares crossed (number): Number of squares (*n* = 16) the pig enters (midpoint of head between the ears) during 1 min observation.	Zones visited (number): Number of different zones (four different arms of the EPM plus center platform) the pig enters (midpoint of head between the ears) during 1 min observation.
Vocalisation	Low grunts (number): Low pitched vocalisation	Low grunts (number): Low pitched vocalisation
Exploration	Explore pen/objects (duration in seconds): Pig makes snout contact with ball, feeder or floor/wall of arena.	Root (duration in seconds): Pig makes contact with floor substrate and makes repetitive nosing movements with snout.

**Table 2:** Terms (2 for each observer) that showed the highest positive and negative correlations with dimensions 1 and 2 of the consensus profile. Figures in brackets indicate the number of observers using that term.

	Positive correlation	Negative correlation
Dimension one	confident (5), curious (5), active (4), exploratory (4), inquisitive (2), bold, interested, relaxed, self-assured	unsure (4), nervous (3), confused (2), fearful (2), hesitant (2), uncertain (2), cautious, frightened, frozen, passive, reluctant, scared, tense, wary, worried
Dimension two	agitated (5), angry (3), frustrated (2), active, annoyed, boisterous, curious, determined, exasperated, irritated, pushy, restless, scared, seeking-reassurance, stressed, tense, upset	calm (7), relaxed (4), quiet (2), comfortable (2), cautious, bored, dull, happy, immobile, laid-back, lethargic, passive, peaceful,
